# Training, teamwork, and a sense of belonging: describing youth sport and research investment

**DOI:** 10.1080/07853890.2025.2521442

**Published:** 2025-07-01

**Authors:** Zachary Yukio Kerr, Lauren V. Fortington

**Affiliations:** a Department of Exercise and Sport Science Injury Prevention Research Center, University of North Carolina at Chapel, Chapel Hill, NC, USA; b University of Sydney, Sydney, Australia

## Introduction

Youth athletes thrive in environments that promote training and development, teamwork, and a sense of belonging [1,2]. The article collection, ‘Injury Prevention in Youth Sports’, showcases the breadth of issues that exist within youth sport that are affected by such factors. These can include serious health issues (e.g. fatalities and cardiac changes [3,4] as well the need to establish a joy in sport and movement that can last a lifetime [5–7].

These same features – training and development, teamwork, and a sense of belonging – are also needed for sport injury prevention research to thrive. In this editorial, we discuss the pressing need for long-term strategy and investment in the next generation of researchers to ensure the future sustainability and advancement of a critical field that directly impacts the health, safety, and well-being of youth athletes globally.

## Training and development

I don’t want to be the next Michael Jordan, I only want to be Kobe Bryant.

- Kobe Bryant

There is no quicker way for a scientist to bring discredit upon himself and on his profession than roundly to declare- particularly when no declaration of any kind is called for- that science knows or soon will know the answers to all questions worth asking…

- Peter Medawar

The article collection, ‘Injury Prevention in Youth Sports’, emphasizes the role of the many mentors that shape the development of youth athletes, including coaches, parents, and administrators [8–10]. Likewise, we as a research community must ensure the continued mentorship of the next generation of researchers. We (the authors of this editorial) have benefitted from mentorship that is engaging, empathetic, and experiential. We have both also experienced leadership that aims to invalidate our own unique identities, ideas, and ideologies, and was instead focused on creating carbon copies of themselves. The former approach has facilitated our individual growth and has motivated us to implement similar mentorship practices with our mentees; the latter approach manifests in sporadic feelings of imposter syndrome stemming from believing that we do not fit in with an established norm.

The development of the next generation of researchers involves growth in both the mentee and mentor. Resolving to stay comfortable in what one knows inhibits personal growth and the development of novel and innovative ideas remains stale and stagnant. Leaders must provide opportunities to a diverse mix of mentees to assist not only the growth and development of the next generation of researchers, but that of the organization’s mission and values. Funders must also be willing to incentivize groups built upon diversity of academic disciplines and backgrounds. Assessment of researchers (e.g. funding, promotion, contribution) would ideally consider their mentorship of the next generation of researchers.

## Teamwork

Sticks in a bundle are unbreakable.

- Kenyan Proverb

Science is a field which grows continuously with ever expanding frontiers. Further, it is truly international in scope. … Science is a collaborative effort. The combined results of several people working together is often much more effective than could be that of an individual scientist working alone.

- John Bardeen

**Figure 1.**
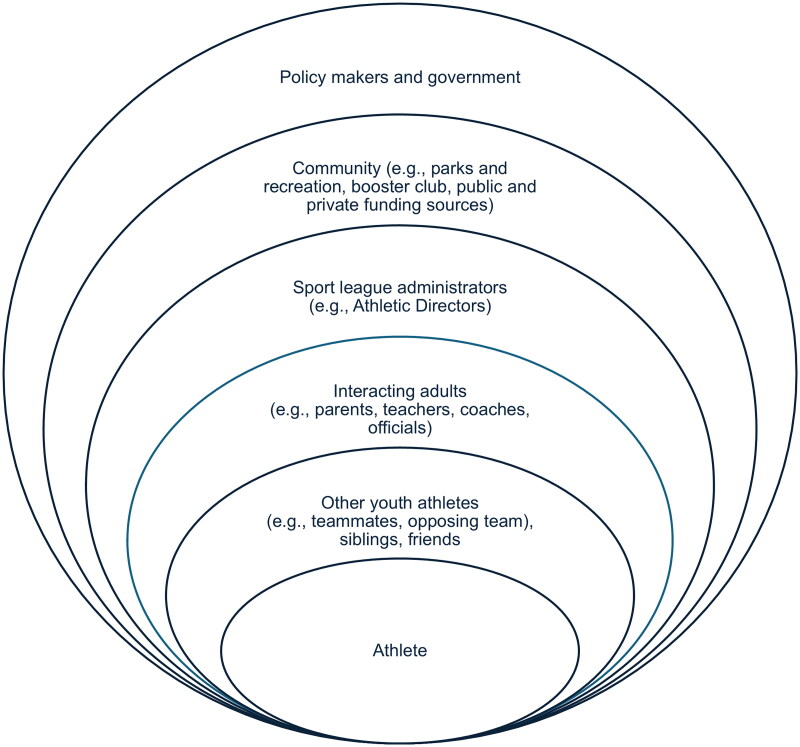
Example of a socio-ecological framework for youth athlete development. Constituents and levels of influence will vary by specific setting.

The athletic triangle [11], comprised of athletes, coaches, and parents, has been used to denote the individuals involved in youth athlete development. However, the organization of youth sports includes many more constituents who are invested not just in athletic development but also safety and success on and off the field. A more encompassing framework, such as that presented in the socio-ecological approach, may be a better fit when considering the range of entities that influence an athlete [12]. Figure 1 provides an example, although the specific types of constituents and levels of influence will vary by each specific setting. To summarize, the constituents that influence a youth athlete include not only their individual beliefs, attitudes, norms, and knowledge, but also the following:

- Teammates and other youth athletes within their social networks, siblings and friends [8]
- Adults that closely work with or alongside the athletes, such as their parents, coaches, teachers and officials [9]
- Youth sport organization administrators and community leaders that may shape access to youth sports [10]
- Policy and legislation related to youth sport safety and associated funding (e.g., park improvements) [13]

The article collection, ‘Injury Prevention in Youth Sports’, covers many aspects of this framework by addressing these factors that help to shape safety and wellness in youth sports. Likewise, as a research community, we must continue to build teamwork within our labs and organizations. Previous research [14] highlights components such as creating shared goals and language.

## A sense of belonging

You cannot change any society unless you take responsibility for it, unless you see yourself as belonging to it and responsible for changing it.

- Grace Lee Boggs

A team isn’t a bunch of kids out to win. A team is something you belong to, something you feel, something you have to earn.

- Gordon Bombay (from The Mighty Ducks film)

**Figure 2.**
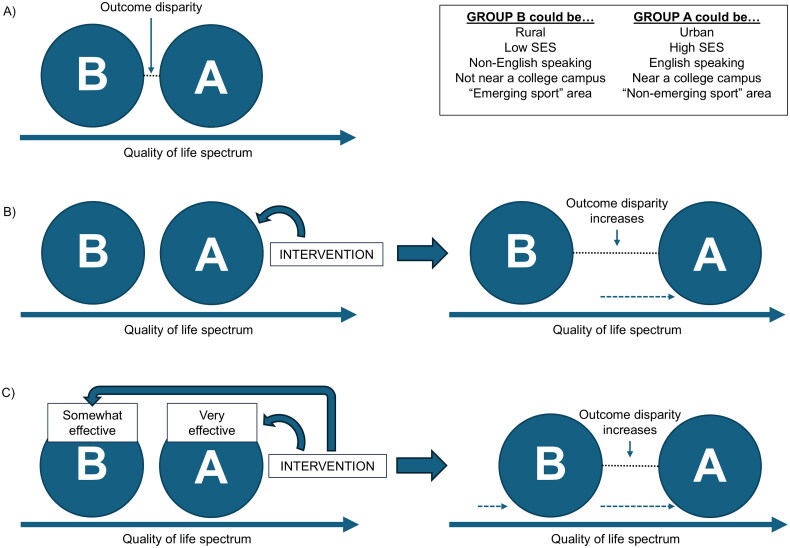
Examples of how limiting research reach may exacerbate health disparities. The quality-of-life spectrum shows Community B with a lower baseline quality of life than Community A, with the gap representing existing disparity. Panel A illustrates this baseline. In Panel B, when interventions reach only Community A (typical when research samples come from higher socioeconomic areas near universities), disparities widen as only one community benefits. Panel C shows that even when both communities receive interventions, disparities may still increase if interventions are implicitly designed for Community A’s context, overlooking Community B’s specific barriers.

In our push for belonging, we must expand what youth sports are examined. In general, research on sport injury prevention is limited in two manners. First, research continues to be dominated by male-centric sports, particularly American Football [15]. However, we must acknowledge the efforts by funding sources with vested interests in the sport, such as the National Football League and the National Collegiate Athletic Association (NCAA). Second, the leagues/organizations chosen to be examined typically are near the researchers and their home institutions, which are likely to be of higher socioeconomic status. Evaluation efforts acknowledge that ‘ideal scientific conditions’ are a first step in the development and examination of injury prevention; nonetheless, we may have reached a plateau in which additional efforts may need to further explore sports outside of the ‘researchers’ comfort zones’ [16]. A continued push for research based on easily accessible samples may create further disparities between the ‘haves’ and the ‘have nots’ [17,18]. Figure 2 illustrates scenarios in which this may occur.

Trust takes time and is necessary to ensure community buy-in. As research continues to integrate social determinants of health metrics in their agendas [19–21], it is important to ensure such work does not further impose barriers between the researchers and their participants. The treatment of data must come with a sense of humanness, and an understanding that ‘every row of data tells a story’ [22].

## Moving forward with resilience and patience

I was taught the way of progress is neither swift nor easy.

- Marie Curie

Resilience is a team sport. You need to have relationships and people to rely on. You don’t do it on your own.

- Stephen Gonzalez

Good research takes time and investment. However, many in the field know that scientists are fighting for strategic ongoing funds as we face being in short supply. This parallels many youth sport organizations where funds are also heavily reliant on the community (e.g. booster clubs, private donations). Communities of lower socioeconomic status may have less such resources available. As a field, we must remain vigilant in continuing this work, while also advocating for increased funding for youth sport injury prevention and safety. Numerous organizations have advocated for athlete safety alongside the benefits of sport participation. And although the scientific community aims to ensure the understanding and declarations of potential competing interests or conflicts of interest, funding from professional sport organizations can be often riddled with concerns that resultant findings are inaccurate or falsified [23].

To ensure that youth sports continue to thrive and provide safe platforms for physical activity, we as researchers must invest in promoting these strengths in our own research. This call requires collaboration, teamwork, and most importantly, patience, as shared goals are developed and enacted through shared ideas and shared research [14]. We appreciate the work of all the scientists involved in this special collection and thank the readers for their interest and attention to the topics discussed.

## Data Availability

Data sharing is not applicable to this article as no new data were created or analyzed in this study.
